# Why I Write for Independent Journals

**Published:** 1908-08

**Authors:** G. Frank Lydston

**Affiliations:** Chicago, Ill., Professor of Genito-Urinary Surgery, State University of Illinois


For Texas Medical Journal.

### Why I Write For Independent Journals.

BY G. FRANK LYDSTON, CHICAGO, ILL.,
Professor of Genito-Urinary Surgery, State University of Illinois.

While attending one of the sections of the A. M. A. at the recent Chicago meeting, I was asked by a certain ethically (?) hyperesthetic medico-literary snob, who industriously seeks for motes in his confrere's eye regardless of the beam in his own, why I wrote articles for "that fellow X's journal." My answer was that it pleased me so to do. Although I am heartily in sympathy with the moral of a certain Rabelaisan story, which, in effect, is "to h—l with the other reasons." I'm going to expatiate, enlarge, amplify, elucidate and—"conflagrate" the theme a bit, earnestly hoping that the multitudinous ultra-ethical self-labeled medico literary perfecto will eventually be told what I have to say. Indeed, I'm sure he will, and, moreover, that he will stop browsing among the thistles of discontent just long enough to gather new notes for his raucous, discordant bray—that bray of narrow-minded, illogical protest wherewith alone he attracts the attention of the professional rank and file to himself and, incidentally, of course, to his literary holy of holies, choked to the brim with intellectual sweepings from other men's garrets.

Obviously, I am not bidding for popularity with certain self-styled journalistic "leaders."

The feature of the better class of independent journals that appeals most strongly to me is the mere fact of their independence in wearing no brand or collar. As matters medico-literary are now trending, the day is not far distant when the average practitioner of medicine will have no medium of expression, no literary representation and no literary pabulum of practical value within the comprehension of the average medical mind. Medicine is fast becoming so scientific, so turgid with "things that ain't so," or which are at least "under suspicion," that the main purpose of medicine, the healing of the sick, bids fair to be lost in the maze of laboratory experimentation and illogical deductions from mentally indigestible "facts"—scientific bricks without straw—from which none but a wizard could build an enduring fabric. What boots it to the practitioner of the cross-roads that there be opsonins and opsonic indices? He has neither the technical training, the appliances nor the time to practically apply them in his daily work. Besides, who knows how soon the opsonins will be gathered to the snows of yester-year?

I fancy I hear the ultra-scientific ones cry, "Let the practitioner of the cross-roads and the hamlet hie him to the post-graduate school and cultivate—at so much a cultivate—'the optic sharp I ween that sees things which are not to be seen.' Let, also, the student of medicine be more thoroughly prepared in things scientific."

As to the post-graduate school, it often makes confusion worse confounded. Abdominal and other special surgeons "made while you wait"; men who entered the mouth of the hungry P. G. school, passed immediately through its short, angleworm-like *primae viae* and promptly tumbled down the back steps with a special course certificate in their hands, have not seldom outheroded Herod—which means that where the haughty professor of the special P. G. course hath slain his dozens, some of his half-baked special students have slain their scores, aye, hundreds.

Once on a time I was asked to respond to the toast, "Post-Graduate Schools." (Be it understood, I myself was a P. G. professor then.) My speech was short and sweet. It was as follows:

"Gentlemen, in response to the toast I will merely relate a story: Mascagni, the great composer, was once visiting New York. Whilst standing at his window in the Fifth Avenue Hotel, he chanced to hear the agonizing shrieks of a hand organ. He listened and to his horror caught the strains of a discordant attempt at his own favorite composition, the *Cavalleria Rusticana.* He rushed madly down the stairs, seized the greasy organ-grinder by the shoulders, shook him and cried, 'You play eet horrible! De tempo ees wrong! Play eet dis a way.' Suiting the action to the word he proceeded to illustrate as best he could on the wheezy old organ. The next day as Mascagni was strolling in the neighborhood he saw a crowd gathered around an organ-grinder who was murdering the *Cavalleria Rusticana.* The great composer approached the crowd, peered through and saw his compatriot industriously working away at the same old organ. On the front of the battered musical relic was a large placard which read, 'Signor Pietro Sylvestre—Pupil of Mascagni.' "

Our medical schools are responding with alacrity to the demand for ultra-scientific training. The *ultima thule* of medical teaching in some quarters apparently is the manufacture of half-educated scientists, not trained physicians. Here is an illustration of some of the brilliant results. I recently had occasion to inquire into the knowledge of materia medica and therapeutics possessed by a recent graduate of a well known school, who, by the way, was one of the ten "Honor Men" in his class:

Question—"What is the botanic name of the plant from which opium is derived?"

Answer—"Poppy, I think."

Q.—"What is papaver somniferum?"

A.—"Poke root."

Q.—"What are the alkaloids of opium?"

A.—"Morphine and atropine."

Q.—"What preparation of aconite would you ordinarily prescribe internally?"

A.—"Why, aconite."

I suggested that the tincture was an eligible preparation, and informed him that there were two tinctures.

Q.—"Which tincture would you give to a child?"

A.—"The tincture of the root, because it's the milder."

Q.—"What dose of the tincture of the root would you give to a child six months old?"

A.—"Oh, about half a drachm every hour."

Q.—"Given the same child and a stimulating expectorant being indicated, what would you give?"

A.—"Carbonate of ammonia."

Q.—"In what dose?"

A.—"Oh, twenty grains every three hours."

Be it remarked that materia medica and therapeutics are taught in the sophomore year in the school from which this gentleman graduated. The treatment of disease is taught before the *raison d' étre* of treatment has dawned on the student's mind. But, this newly-fledged graduate knows a lot about the embryology of the chick—he had watched it for weeks—the nervous anatomy of the frog, neurons, opsonins and things—which knowledge is not likely to save from massacre the first hapless infant he treats.

The independent medical journal meets the demand of the everyday practitioner who wants to know "what to do." The self-styled high-class medical journal—and there is really only one "high-class" journal, you know, which is climbing so high that its head looks from below very like that of a pin—often gives him a stone when he asks for bread. He seeks for light on the treatment of disease, and on looking over the menu card presented by the "most high," he finds such things as "My Last Thousand Cases of Excision of the *Calamus Scriptorius*," "My New Postural Method of Catheterizing the *Iter a tertio ad quartum ventriculum*," "The Opsonic Index in the Care of the Second Bicuspid," etc., and editorials in which the mantle of dignity conceals vast intellectual abysses. In despair he turns to that cemetery in which so many fond therapeutic hopes lie blasted and buried under tons and tons of therapeutic nihilism, Osler's *Practice*—and still he finds no balm in Gilead. And then he turns to the independent journal and is consoled—which is a blessing, e'en though he be sometimes cajoled into belief in things unsubstantial. And the proof of the pudding is that thousands upon thousands of doctors buy and read the very journals upon which the "lily whites" of medical journalism frown so blackly.

The practitioner knows full well that his patient wants something more than a diagnosis. When that has been made the ultra-scientific (?) doctor may be satisfied, but the afflicted one, like Chimmie Fadden, says, "That's all right, Doc. But, what t'ell?" And just because medical science has whored too much after strange and weird scientific gods and has not told the patient "What t'ell?" quackopathy flourisheth in the land and the high priestess of modern humbug, Mary Baker G. Eddy, hath built sundry and multitudinous gold and marble palaces like unto the temple of Solomon. And, by the same token, any quackopathy which is not intrinsically murderous and is a harbinger of hope, is often better than acute paranoia anti-therapeuticum Baltimorensis, which sets forth in dismal colors the disease, and threads the gloomy picture with not one silver strand of hope. Given a little hope, even though it be ill-founded, and the patient is likely to be better off than in the possession of the most accurate diagnostic knowledge which a pessimistic, ultra-therapeutic nihilist can offer.

Be not vainglorious and puffed up, O ye macrocephalic ultra-"ethical," ultra-"scientific" Philistines. Time was when Hahnemann was the medical anti-Christ, the black beast of medicine. And here we are in the midst of an organo-therapy which suggests that the sometimes befuddled Samuel builded wiser than he knew. Auto-vaccination suggests to me that my unfavorable opinion of my old hospital chief's—Dr. Carnochan's—prescription of triturated *chancrum durum* for syphilis, expressed nearly thirty years ago, might have been a bit hasty.

Do you know, brethren, that my admiration is daily excited by the magnanimity of the Homeopath? And he excites my sympathy, also. Of late years he has been tumbling over himself to get into our band wagon. If he had only bided a wee! We are fast climbing into his. We call things by different names, but—"a rose by any other name would smell as sweet." Auto-vaccination and *similia similibus* do not make good rhyme, but they smell an awful lot alike—so much so that I have taken the sole surviving copy of my lecture on "Homeopathy and Its Congeners," delivered a quarter of a century ago, and hidden it under my study floor. And now daily am I reminded of Poe's "Tell Tale Heart" and of his Raven's doleful "Nevermore."

The ultra-scientific one who does not overmuch believe in treatment and recognizes naught but the scalpel and hemostatic forceps sometimes marvels that any one could condescend to read, much less contribute to our independent journalistic media of medical expression. "Nothing in drugs," he wails; "send 'em to me and I'll cut 'em." He forgets that modern science has not yet conquered the lay aversion to the knife, nor the honest practitioner's belief that, after all, the knife is often a confession of our limitations and weakness. And there is much in the training of the experienced practitioner which inspires him with therapeutic hope in a vast number of the ills of the flesh. By drugs he can produce anesthesia, local or general, relieve pain, produce sleep, stimulate or depress the circulation, allay nervous irritability, aid digestion, relieve constipation and hepatic torpor, produce emesis, diaphoresis and diuresis, antidote malaria, and cure syphilis. What wonder that he has confidence in drugs *per se* whilst rather skeptical of our knowledge of them? "There must be a remedy. If I only knew"—is a brow-contracting reflection familiar to the conscientious practician. And so long as there are sick ones to heal so long will he search for remedies—and so long will he read and believe in the literature that offers therapeutic hope.

While writing this I recall with some amusement the comparative merits of the gentleman who asked me why I wrote for X's journal and of X himself and his staff of journalistic colaborers. Nature did much for both men, but X made his own opportunities while fate fairly showered them on his critic. Death's remorseless scythe swept out of the critic's path life after life that stood between him and professional position. What fate did not accomplish, great wealth and high social position wrought—and wrought well. What he is was made by the hand of fate—albeit I grant that the original material was pretty good stuff. As for X, the good stuff in him was molded by the hand of X himself under the stimulus of grim necessity. And now he owns and operates a medical journal which reaches, instructs and holds more of the rank and file of the medical profession than most extant publications. Associated with him are two of the wisest and best therapists that this country has ever produced. And the journal is doing good. It is not always right and is sometimes a bit "woolly," but in the main it is doing good by giving the average practitioner not only instruction, but a medium of expression.

Apropos of the snobbish question, "Why do you write for X's journal?" I myself have something of an eye to the "medium of expression" end of medical writing. If I have anything of value to say, I fancy that it does the greatest good to the greatest number in the journal that reaches the largest number of average general practitioners. Moreover, there's where it does *me* the most good—and be it remarked, I am not one of those who profess to be writing "for the good of humanity" first, last and all the time. I believe that the product of my pen which does the profession and humanity the most good, is the stuff that is most likely to do me good, and *vice versa*. The hypocrisy and conceit of the medico-literary snob with a heaven-born "message" make me seasick. The pinheaded egotist wasting midnight oil in compiling ideas—or "facts," rather, for an idea would addle his composition—from other men's work for his message to an eagerly expectant scientific world is a spectacle for gods and men! And what shall we say of the toiling brother, primarily infertile of brain and who, dreading the pains of even the mechanical operation of literary parturition, merely affixes his name and manifold unearned titles to a compilation prepared by some poor devil of a medico-literary hack? I once heard vociferous and earnest applause at an overflowing meeting of a great medical society rendered a paper which had but one original line in it—the name of the author—and that was composed by his parents and written by his typewriter. Alas! poor literary Adam. And this is the sort of stuff that fills some of our ultra "high-class" journals to overflowing.

Once on a time an inky-handed pirate on the literary high seas published as his own in a high-class Eastern journal an article of mine written and published more than fifteen years before. He did not alter a line—scarcely even a word of it. Yet it was taken as original stuff by the aforesaid journal. The literary buccaneer is now editing an ultra-classy medical journal to which none but the literary elect among authors need apply. Those who are interested will find the above matter well ventilated in the *Journal of the A. M. A.* several volumes ago in an article by me entitled, "How to Write a Medical Article—A Plea for Plagiarism,"—the which is a sad commentary on some "high-class" journals and their recondite editors.

"Humanity?" "Science?" For the pinheads, "humanity is I, writ large," and science is an incoherent jumble of indigestible "facts" and dangerous excreta from the intellectual bowels of threadless wanderers in up-to-date laboratory mazes. But the flies on the chariot wheel, the "ultras," do not "'fess up," so let's pretend that we believe their sniveling hypocritic "science and humanity" drivel, whilst contritely acknowledging the hope that whatever we do for science and humanity may incidentally do some good to ourselves—and some more *vice versa.*

One of the principal objections of the "super-perfects" in medical literature offer to the independent journal is the character of its advertisements. Time was when that journalistic mentor, the *Journal of the A. M. A.,* would take almost any old thing in the way of a paid ad. Wherein lies the change of front? Is it a matter of conscience, or a fat-bellied prosperity that no longer needs or craves the flesh pots of Egypt? Once upon a time this ad appeared in its columns: "Wanted—A gentleman, past middle life, who has been incapacitated by a surgical operation for the performance of his conjugal duties, would like to meet a lady similarly situated. Address, No. 1001, Journal office." What has happened? The same editor—salaam, please—is in charge, and the trustees haven't changed all around. Item: They couldn't; the political machinery is too perfect. This is not a kick, but a compliment. Once more, salaam, O ye faithful.

No official notice was taken of the ad above mentioned, so I infer that it "went," as a matter of course. Really, somebody must recently have injected a large dose of ultra-ethical serum into the veins of the reigning medical dynasty. The tin gods will please sit up and take notice. They can not quite hide their light of goodness under the bushel of modesty.

But the reigning dynasty has not yet ceased straining at gnats and swallowing camels. It is still possible to advertise in its columns "legitimate" preparations that would under no circumstances be admitted into its reading matter. Under which king, Bezonian —Hypocrisy or Flesh Pots?

A special feature of the independent medical journal which commends itself to me is the possibility of individual expression in its editorial pages. Vigorous independent thought trenchantly expressed is what the medical man most needs. And the thought expressed should not always be medical dry bones. Medicine is broad. It should embrace things literary, political and sociologic. Take the editorial columns of the independent medical journals away from him, and the overworked practitioner will be in a bad way for intellectual pabulum. The editor of an official "society organ" who should venture to express himself in terms stronger than a literary milk shake couldn't hold his job for twenty-four hours. Take away editorial independence and what would the organizers of a professional monopoly or a medico-political trust have to fear? What check would there be on their system? Why, they would not meet even criticism of any degree of potency.

The leaven of consolidation, unification and trustification is working most potently. By and by, the firmament of American medical literature will contain naught but a central literary sun and his satellites, the "State" journals. The independent journal that has been the representative at court and the great educator of the medical rank and file will be no more—and the rank and file will die of intellectual inanition, starved to death on the mental breakfast foods prepared by the great medical trust whose bat-like wings are already casting baleful shadows over the profession. The average practitioner will hunger and thirst for intellectual pabulum—and he will get the shavings and gelatine broths dispensed by the hierarchy. The handwriting is on the wall.

Oh, for the pen of a Moliere or the merciless and clever literary caustic of a Voltaire! Nay, I do but overglorify my theme; the drollery of a Josh Billings, an Artemus Ward or any wearer of the cap and bells would be about the right size of shot to fire at this particular bird.

If only I had the genius—big or little—what a picture I would now paint on my reader's mental screen. The incident I am about to relate apropos of ethics in high places combines more diverse elements of sincerity, hypocrisy, buncombe, drivel, humor and pathos than anything I ever heard of:

A certain medical man, big, brainy, square-toed and altogether lovable, felt called upon to explain to a certain official medical body the publication of his picture amid a group of other distinguished physicians in a certain newspaper. "I called at the newspaper office," he explained, "and demanded the picture. I was informed that it was the property of the paper. I find that I have no legal means of redress," etc., etc. This in face of the fact that the eminent gentleman's name has appeared in connection with the treatment of prominent citizens oftener than that of any physician in the country. Why, I once saw it in two or three separate places in the same paper! Not long since his attendance on a certain multi-millionaire and his large fee for the same were newspaper talk by the column for days and weeks. Oh, fudge! old man; why didn't you tell 'em to go plumb straight to h—l? Manly expression—and you are a man, if ever there was one—is cheaper and thicker—aye, and more beautiful—than whitewash of the auto variety. Why should not the eminent medical man be as much public property and of as much public interest as though he were a lawyer or a statesman?

The struggle of the medical babies to keep their erythematous rear elevations covered with the ethical garments inherited from our medical daddies is agitating to one's sense of humor. No use; our professional daddies didn't employ wool soap! Still less did they use good horse sense—if they had, they would have realized that the medical man is a creature of his environment and must adapt himself to it or be a social anachronism and a political nonentity.

Moral—Don't be a clam merely because the paleozoic senilescents of a dead and gone medical age were contentedly stuck in the fossiliferous mud on the shores of the ocean of progress. Let the dead past bury its dead—and bury it deep, and—let us not often open the doors of our ethical museums.

It has occurred to me that the ambition of the doctor to own and operate a medical journal is conducive to the best interests of the profession. The medical editor has in general stood for what is best in medicine. He has often gone astray, it is true, and has sometimes pandered to the proprietors of worthless or doubtful drug preparations, but on the whole the profession has benefited by the influence of the independent medical editor. He has been our watch dog in a way, and while by no means perfect—he is human, you know—has been a pretty creditable part of the body professional. Where he has made a living out of his journal he has been useful by demonstrating a bread and butter outlet for the energies of medical men, and we have, alas! only too few such resources for physicians.

Had I ever wavered in my opinions as to the ethics of contributing to independent medical journals my faith and courage would have been restored by something I saw a few short weeks ago in a journal which the ethical ultras regard so unfavorably that they throw an autotoxic fit and roll up their eyes like a dying jack rabbit whenever they hear it mentioned. It was an article by "Saint George," of Philadelphia. And, *mirabile dictu,* it was headed by his picture! Think of it—the peerless St. George, the erstwhile *arbiter elegans* of medical literature, slayer of ethical dragons and mastodonic medical hypocrites and humbugs, peerless knight of the medical ink pot, had an article and picture in a journal owned and controlled by a manufacturer of pills and "sich!" And—oh, joy! I had an article—with picture—in the same issue of that proscribed magazine. Shall it longer be said that Munyon with his awe-inspiring index sweeps unopposed on his majestic way? No, a thousand times no.

Verily, "the world do move."

---

For Texas Medical Journal.

### Evolution.

(With apologies to Langdon Smith.)

When you were a lobster and I was a peach
  In our youth's roseate time,
And side by side on Broadway's tide
  We blew the froth from the festive stein,
Or staggered with many a stag
  Through the haunts of dissolute men,
I wasn't next to the ways of life
  And you were a sucker, even then.

Heedless we lived, and heedless we loved,
  And heedless, at last, we died;
And deep in a hole in the Potter's Field
  They planted us, side by side.
The town rolled on with her hot old time,
  And Broadway heaved amain;
But we caught no breath from the womb of death,
  And we never peeped again.

So we can't take lunch at Delmonico's,
  'Tis a joint that's quite out of reach;
And we'll not drink anew to the time when you
  Were a lobster and I was a peach.

—LYDSTON.

### Climate and Outdoor Life in Tuberculosis.

P. Paquin, Asheville, N. C., says that in the use of climate in the treatment of tuberculosis we are chiefly concerned with its effects on nutrition, which is the basis of resistance against the tubercle germs and its toxins. It should be premised that there is no perfect climate, and often too much is expected by both physicians and patients. We need also to classify patients as regards climatotherapy, and in the first class Paquin would put the advanced consumptives of all types, and in general the physically depressed to a grave degree under any form of tuberculosis, save, perhaps, tuberculosis of the brain. Generally, these patients seem to do better at higher and dryer altitudes than at lower ones, or where moisture is great. In the second class he would place the dyspeptic, the so-called pretuberculous cases, the incipient developments and possibly all prosoftening stages and the early mixed infections. These patients may do well almost anywhere in pure air, but, other things being equal, do much better at medium altitudes, and may do well at high altitudes. It is largely an individual question, the most reasonable solution being to send them where, if a cure follows, they can return home and live with comparative safety. Paquin goes into considerable detail in regard to the effects of temperature, sunshine, humidity, pressure, etc., their benefits and disadvantages. Heat and moisture combined are depressing and high winds, with dust or otherwise irritating, cold blast winds, raw chilly winds, hot moist winds, etc., are to be avoided. Cold air in a fairly dry locality is beneficial, but is the reverse in most regions. Information on these points is necessary in selecting a preventive or curative climate for tuberculosis. Intelligent management of outdoor life is also essential. The patients need instruction in how to live and sleep in the open air with safety. Special consideration should be given to the matter of getting up from bed in cold weather, and Paquin describes a device for sleeping in the open air safely in any weather, adopted by him at the Asheville-Biltmore Sanitorium. In conclusion, he calls attention to the still existing imperfections in our knowledge of climatology and our consequent blunders, and the need of systematic study of the subject by the profession.—*Journal A. M. A.*

---

A Dry Doc.—It is told of Mark Twain that during a conversation with a young lady of his acquaintance he had occasion to mention the word drydock.

"What is a drydock, Mr. Clemens," she asked.

"A thirsty physician," replied the humorist.—*Carolina Medical Journal.*

---

"What you want to do," said the druggist, as he handed the old darkey the medicine, "is to take a dose of this after each meal." "Yes suh," was the reply, "an' will you please tell me whar I gwine ter git de meals?"—*Atlanta Constitution.*

